# Pepper growth promotion and biocontrol against *Xanthomonas euvesicatoria* by *Bacillus cereus* and *Bacillus thuringiensis* formulations

**DOI:** 10.7717/peerj.14633

**Published:** 2023-01-24

**Authors:** Jared Hernández-Huerta, Patricia Tamez-Guerra, Ricardo Gomez-Flores, Ma. Carmen E. Delgado-Gardea, Loreto Robles-Hernández, Ana Cecilia Gonzalez-Franco, Rocio Infante-Ramirez

**Affiliations:** 1 Facultad de Ciencias Agrotecnológicas, Universidad Autónoma de Chihuahua, Chihuahua, México; 2 Facultad de Ciencias Biológicas, Universidad Autónoma de Nuevo León, San Nicolás de los Garza, Nuevo León, México; 3 Facultad de Ciencias Químicas, Universidad Autónoma de Chihuahua, Chihuahua, México

**Keywords:** Antagonism, Pepper, Bacterial spot, Bactericidal effect, Plant growth promoting bacteria

## Abstract

**Background:**

*Bacillus* genus has been used in horticultural crops as a biocontrol agent against insect pests, microbial phytopathogens, and plant growth-promoting bacteria (PGPB), representing an alternative to agrochemicals. In particular, *B. cereus* (Bc) and *B. thuringiensis* (Bt) have been studied for their fungicidal and insecticidal activities. However, their use as biofertilizer formulations and biocontrol agents against phytopathogenic bacteria is limited.

**Objective:**

To evaluate Bc and Bt formulations as PGPB and biocontrol agents against the bacterial spot agent *Xanthomonas euvesicatoria* (Xe) in greenhouse-grown chili peppers.

**Methods:**

Bc and Bt isolates obtained from soil samples were identified and characterized using conventional biochemical and multiplex PCR identification methods. Bioassays to determine Bc and Bt isolates potential as PGPB were evaluated on chili pepper seedlings in seedbeds. In addition, formulations based on Bc (F-BC26 and F-BC08) and Bt (F-BT24) strains were assessed as biofertilizers on pepper, under controlled conditions. Furthermore, *in vitro* antagonism assays were performed by confronting Bc and Bt isolate formulations against Xe isolates in direct (foliage) and indirect (resistance induction) phytopathogen biocontrol assays on pepper plants, which were grown under controlled conditions for 15 d after formulations treatment.

**Results:**

Isolates were identified as Bc and Bt. Formulations significantly improved pepper growth in seedbeds and pots, whereas *in vitro* bioassays demonstrated the bactericidal effect of Bc and Bt strains against Xe isolates. Furthermore, assays showed significant plant protection by F-BC26, F-BC08, and F-BT24 formulated strains against Xe.

**Conclusion:**

Results indicated that F-BT24 and F-BC26 isolates formulations promoted pepper growth and protected it against *Xanthomonas euvesicatoria*.

## Introduction

Pepper (*Capsicum annuum* L.) is one of the most cultivated and consumed vegetables worldwide, after potatoes and tomatoes (García-Gaytán et al., 2017; Lozada et al., 2022). In Mexico, pepper crop importance relies on its economic and social input due to working labor demand, extensive consumption, and high profitability (Bobadilla-Larios et al., 2017; Agri-Food and Fisheries Information and Statistics Service of Mexico (SIAP), 2021). In 2021, pepper production was 3,086,742.28 tons, with a production value of 1,532 million US dollars, thus placing Mexico as the second producer globally (Agri-Food and Fisheries Information and Statistics Service of Mexico (SIAP), 2021). However, abiotic and biotic factors may reduce its yield. *Xanthomonas euvesicatoria* (Xe) is the bacterial spot causal agent and may lead to crop yield reduction by up to 66%, with losses of up to $7,500 US dollars per hectare (Jones et al., 2004; Sharma & Bhattarai, 2019; Agri-Food and Fisheries Information and Statistics Service of Mexico (SIAP), 2021).Therefore, administration and optimization of available resources are required for commercial production, including labor and agrochemical inputs (mainly fertilizers, pesticides, fungicides, and bactericides) (Macías, Grijalba & Robles, 2012; Šević et al., 2019; Osdaghi et al., 2021). Because of agrochemicals adverse effects on the environment and animals’ health, research on ecological and low-cost alternatives for this crop management has been conducted. The use of selected microorganisms in horticultural crops is an effective alternative to agrochemicals for pest and disease control, as well as for fertilization purposes (Waguespack, Bush & Fontenot, 2022). In this regard, strains of the genus *Bacillus* isolated from soil or phyllosphere have been used as biocontrol agents against insect pests and phytopathogens, and as plant growth-promoting bacteria (PGPB) (Saxena, Karthikeyan & Rajawat, 2017; Kashyap et al., 2019; Tiwari, Prasad & Lata, 2019; Mahapatra, Yadav & Ramakrishna, 2022). *Bacillus* spp. such as *B. amyloliquefaciens*, *B. cereus*, *B. pumilus*, *B. subtilis*, *B. thuringiensis* (Bt), and *B. velezensis* colonize plant roots, producing exopolysaccharides, siderophores, or phytohormones, as well as solubilize phosphorus or fix nitrogen (Gupta et al., 2021; Chaudhary et al., 2022). *Bacillus* strains are also known to induce plant defense response against phytopathogens, improving growth and yield in several crops (Yanti & Nasution, 2017; Tunsagool et al., 2019). Studies on *B. cereus* and *B. thuringiensis* are related to their fungicidal and insecticidal activity. However, reports on using *Bacillus* strain formulations as biofertilizers and biocontrol agents against species of *Xanthomonas*, particularly in pepper, are scarce.

*B. thuringiensis* has been shown as PGPB in cabbage, pepper, lettuce, and tomato crops, with significant increases in several vegetative development parameters (Praça et al., 2012; Abdeljalil et al., 2016; Hyder et al., 2020; Jo et al., 2020). Applying Bt plus mycorrhizae confers drought stress tolerance and improves lavender growth (Armada et al., 2016), whereas its use in consortia with *Rhizobium leguminosarum* improves peas and lentils growth (Mishra et al., 2009). Bt has also been reported to accelerate flowering in soybeans and tomato, increasing their yields (Yanti, Habazar & Resti, 2017; Batista et al., 2021). In addition, *B. cereus* has been demonstrated as PGPB in pea, potato, pepper, tomato, and soybean crops (Kurabachew & Wydra, 2013; Zhang et al., 2019; Baliyan et al., 2021; Sahile et al., 2021; Kashyap et al., 2021; Shin et al., 2021). Studies have shown that *Bacillus* strains participate in inducing systemic responses (ISR) in plants and controlling several microbial diseases (Shafi, Tian & Ji, 2017; Miljaković, Marinković & Balešević-Tubić, 2020; Samaras et al., 2021; Zhou et al., 2021). The indirect biocontrol of *Xanthomonas* spp. by ISR has been reported after applying *Bacillus* spp. in various crops. In this regard, El-Wakil & Essa (2020) found a significant decrease in barley bacterial blight after applying *B. subtilis* and *B. thuringiensis* on barley seeds and soil against *X. campestris.* Similarly, Islam et al. (2019) reported the control of *X. citri* subsp. *citri* in citrus by *B. thuringiensis* TbL-22 and TbL-26 application, whereas Rumbiak, Habazar & Yanti (2018) demonstrated the biocontrol of soybean blight (caused by *X. axonopodis* pv *glycines*) with Bt. Moreover, Yang, Yu & Ryu (2009) found that disease symptoms decrease after applying *B. cereus* BS107 to pepper plants in the control of *X. axonopodis* pv*. Vesicatoria*, whereas Chandrasekaran et al. (2017) reported the control of *X. campestris* pv. *vesicatoria* in tomato seedlings after *B. subtilis* CBR05 application. Despite these benefits, limited formulated products based on *B. cereus* are available as biofertilizers (Azizoglu, 2019). However, *B. thuringiensis* reports as PGPB and biocontrol agents are scarce. Thus, the present study aimed to evaluate Bc and Bt formulations as PGPB and biocontrol agents against bacterial spot by Xe in greenhouse-grown chili peppers.

## Materials & Methods

Microbiological culture media, reagents, and biochemical test kits were purchased from Sigma-Aldrich Química de México (Toluca de Lerdo, MX), unless otherwise specified. Molecular biology assay reagents were obtained from Promega Corp. (Madison, WI). Field experiments were approved by the Fundación Produce, Chihuahua, A.C.

### Samples collection

Soil samples were collected from 30 chili pepper commercial lots from pepper producers in Meoqui, Chihuahua, MX (28°23′23″N, 105°37′25″W), who are current and former members of the Fundación Produce Chihuahua, A.C. Sampling was performed by selecting five diagonal points per lot, and soil was collected at a depth of 20 cm at each point (Ha, 2014). Samples were then placed in new zip-lock plastic bags, labeled, placed in a cooler, and transported to the Applied Microbiology, Plant Pathology, and Postharvest Physiology Laboratory (MAFFP) of the Autonomous University of Chihuahua (UACH), where they were kept at 4 °C, until use.

### *Bacillus* strains isolation and storage

*Bacillus* strains isolation was performed according to the method adapted by Astorga-Quirós et al. (2014). In brief, one gram of soil from each sample was placed in a test tube containing nine mL of sterile saline solution (0.85%) and covered with a lid. Tubes were then shaken for three minutes with a vortex (Daigger Vortex-Genie 2; Scientific Industries Inc., Bohemia, NY, USA) set at speed 3. Next, tubes were placed in a water bath at 90 °C for 10 min, after which serial microdilutions were prepared in microplates, according to Chen, Nace & Irwin (2003) and Lugo et al. (2012). For this, 100 µL of soil suspension were taken from each tube and placed in the first series of microplate wells (stock sample), and subsequent wells were filled with 180 µL of sterile saline solution (0.85%). Next, 20 µL of the stock sample were transferred into one well of the second series, shaking in circles with the micropipette tip to have the first dilution. From this, 20 µL of the sample were transferred into the third series of wells, repeating the previous step, until obtaining a dilution of 1 × 10^6^ (Chen, Nace & Irwin, 2003). Dilutions were then transferred to Petri dishes containing nutrient agar medium (NA; BD Difco Laboratories, Sparks, Maryland, MD, USA) using a replicator (Lugo et al., 2012) and incubated for 24 h at 28 °C in a Lab-Line Imperial III incubator (Fisher Scientific, Dallas, TX, USA). Developing colonies were characterized as typical *Bacillus* genus based on the size, shape, elevation, and texture (Calvo & Zúñiga, 2010), and were isolated in NA medium and stored at −20 °C in a 40% glycerol solution in distilled water, until use.

### Phenotypic identification

Presumed *Bacillus* isolates were subjected to standard biochemical and physiological tests (Shaad, Jones & Chun, 2001). Gram stain (Hycel^®^, Zapopan, Jalisco, Mexico), KOH tests, and Schaeffer-Fulton stain were also performed to determine endospores presence (Mormak & Casida, 1985). The hanging drop motility test (Vázquez et al., 2011) was used to determine the motility of an isolate.

### Molecular identification

PCR was implemented to identify *Bacillus* species, using specific primer pairs for *B. anthracis*, *B. cereus*, *B. licheniformis*, *B. mycoides*, *B. subtilis,* and *B. thuringiensis* (Table 1) (Park et al., 2007; Sadeghi et al., 2012). DNA extraction was performed using the modified phenol-chloroform method (Bardakci & Skibinski, 1994). For this, 1.5 mL of each bacterial isolate after 24 h growth in nutrient broth (NB) (BD Difco Laboratories) was centrifuged at 12,000 rpm for 5 min at 4 °C, and the supernatant was discarded. Next, 100 µL of 10% sodium dodecyl sulfate, 100 µL of 5 M NaCl, and 100 µL of 10% cetyltrimethylammonium bromide were added, after which the mixture was homogenized in a vortex at 3,400 rpm for two minutes (VX-200, Labnet International, Inc., Edison, NJ, USA). This mixture was then incubated at 65 °C in a water bath for 10 min, followed by the addition of 750 µL of a phenol:chloroform:isoamyl alcohol (25:24:1) mixture, stirred in a vortex at 3,400 rpm, and centrifuged at 12,000 rpm for 5 min at 4 °C. The upper aqueous phase was carefully collected with a pipette in a new tube. For DNA precipitation, 500 µL of isopropanol were added to the supernatant, shaken at 2,000 rpm, and placed in the freezer at 20 °C for 24 h. This solution was centrifuged at 12,000 rpm for 10 min at 4 °C, and the supernatant was discarded. Next, the precipitate was washed twice with 300 µL of 70% ethanol, centrifuged for 10 min, dried at 25 °C for 30 min, suspended in 50 µL of Tris-EDTA (TE) buffer (10 mM Tris–HCl and 1 mM disodium ethylene diaminetetra-acetic acid (EDTA) solution at pH 8), and stored at −20 °C, until use.

**Table 1:** Primers used for *Bacillus* spp. identification^1^.

Species	Primer	Sequence (5′–3′)	Amplicon size (bp)
*B. cereus*	BCGSH-1F	GTG CGA ACC CAA TGG GTC TTC	400
	BCGSH-1R	CC T TGT TGT AC C AC T TG C TC	
*B. anthracis*	BASH-2F	GGT AGA TTA GCA GAT TGC TCT TCA AAA GA	253
	BASH-2R	ACG AGC TTT CTC AAT ATC AAA ATC TCC GC	
*B. thuringiensis*	BTJH-1F	GCT TAC CAG GGA AAT TGG CAG	299
	BTJH-R	ATC AAC GTC GGC GTC GG	
*B. cereus*	BCJH-F	TCA TGA AGA GCC TGT GTA CG	475
	BCJH-1R	CGA CGT GTC AAT TCA CGC GC	
*B. mycoides*	BMSH-F	TTT TAA GAC TGC TCT AAC ACG TGT AAT	604
	BMSH-R	TTC AAT AGC AAA ATC CCC ACC AAT	
*B. licheniformis*	AY185898-F	CTGGGGGACATGCTGATCCGCA	497
	AY185898-R	AAGTCCGGATGGGCGGCACACA	
*B. subtilis*	AJ539133-F	TTTACGATGGCGTTCAGCAAC	744
	AJ539134-R	GGAAGTGCCTTCATTTCCGGCT	

**Notes.**

1 Park et al. (2007); Sadeghi et al. (2012).

Samples DNA concentration was adjusted to 5 ng/µL with a basic Eppendorf BioSpectrometer (Eppendorf do Brasil Ltda., Mexico City, Mexico). We used 25 µL samples in the PCR reaction mix, including 15.5 µL of sterile water, 5 µL of 5X PCR buffer, 1.5 µL of 25 mM MgCl_2_, 1 µL of 25 mM dNTP, 1 µL of each primer (10 µM stock concentration), 1.5 µL of DNA at 5 ng/µL, and 0.2 µL of 5 U/µL Taq DNA polymerase. PCR reactions were performed using a thermocycler (Mastercycler model 5333; Eppendorf AG, Hamburg, Germany). The amplification program consisted of an initial denaturation cycle at 94 °C for 5 min, 30 denaturation cycles at 94 °C for 30 s, and 63 °C for 30 s, an extension at 72 °C for 30 s, and a final extension cycle at 72 °C for 5 min, followed by an initial denaturation cycle at 94 °C for 5 min, 35 cycles of denaturation at 94 °C for 5 min, annealing at 64 °C for 1 min, extension at 72 °C for 1 min, and a final extension cycle at 72 °C for 7 min (Park et al., 2007; Sadeghi et al., 2012). Results were analyzed by 1.5% agarose gel electrophoresis, using 1 X ethidium bromide as intercalating agent for 120 min and visualized in a GenLogic 200 photodocumenter (Kodak, New York, NY, USA).

### Pepper plants growth promotion and formulations

*Bacillus* activity as PGPB in pepper plants was evaluated in two stages, under greenhouse conditions (27 °C ± 2 °C and 75% relative humidity (RH)). In the first experiment, we evaluated pepper seedlings growth promotion (seedling emergence) by 22 *Bacillus* strains. In a second experiment, three formulations were prepared with selected isolated identified and coded as F-BC26 for *B. cereus* strain CBC26, F-BC08 for *B. cereus* strain CBC08, and F-BT24 for *B. thuringiensis* strain CBT24. They were grown in 100 mL of tryptone soy broth (TSB) at room temperature for 72 h, under continuous shaking at 120 rpm. After incubation and sporulation, cultures were placed in a water bath at 90 °C for 10 min to eliminate other non-forming spore bacteria, present as contaminants. Next, the culture was centrifuged at 9,000 rpm and suspended in 10 mL of 1X phosphate buffered saline (PBS) solution (Fisher Scientific), which was inoculated in one liter of TSB and incubated at room temperature for 24 h at 120 rpm.

As previously reported by Tamez-Guerra et al. (2000), formulations were prepared for microgranules production by spray drying (Niro Mobile Minor 2000; GEA, Munich, Germany), using the composite formula. Bacterial cultures were used to inoculate 4 L of TSB in a 7 L flask and incubated at room temperature for 72 h at 120 rpm. Cultures were then mixed with one kilogram of nixtamalized corn flour (Maseca, Molinos Azteca, S.A. de C.V., Guadalupe NL, México), previously dissolved in 5 L of purified water, and kept under stirring. Next, 100 mL of cottonseed oil, 200 mL of Inex A, and 5 L of culture were added, followed by 40 g of CaCl_2_ and 300 mL of isopropyl alcohol. The final volume was processed in the spray dryer, with a turbine pressure of 1.5 bar, an inlet flow of 55 mL/min at 200 °C, and an outlet temperature of 80 °C ± 3 °C, after which microcapsules were prepared by spray-drying (Tamez-Guerra et al., 2000). Formulations were then stored in plastic bags at 4 °C, and CFU was determined.

Formulations were evaluated in pepper seedlings, seedbeds, and potted plants (seedling emergence stage and vegetative stage). The bacterial mixture as consortium was not included as treatment because, in previous experiments, they did not show differences from their individual effect (data not shown).

#### Pepper seedlings growth promotion by Bacillus spp. in the seedling emergence (first experiment)

This experiment was implemented on jalapeño pepper seedlings (*Capsicum annuum* var M, Southern Star Seeds S. de R.L. de C.V., Mexico City). There were grown in 20-cavity polystyrene trays filled with sterile horticultural perlite (1 h at 120 °C and 15 lb/in^2^ pressure). Seedlings were watered every third day with a nutrient solution composed of 5.40 milliequivalents/liter (mEq/L) KNO_3,_4.40 mEq/L NH_4_NO_3_, 2.60 mEq/L Ca(H_2_PO_4_)_2_, 1.00 mEq/L MgSO_4_, 8 ppm Fe (EDTA-Fe 6%), 1 ppm boric acid, and 12 ppm Fetrilon Combi^®^ (9% MgO, 3% S, 4% Fe, 4% Mn, 1.5% Cu, 1.5% Zn, 0.5% B, and 0.1% Mo) adjusted to pH 5.5 and electrical conductivity (EC) of 1.3 milliSiemens (mS)/cm (Otazu, 2010).

Twenty-two *Bacillus* suspensions were prepared by cultivating bacteria in NB for 72 h at 28 °C, after which they were centrifuged at 7000 rpm for 10 min at 4 °C. Next, the supernatant was removed, and the resulting pellet was suspended in a sterile saline solution 0.85%. Suspensions were adjusted to 1 ×10^8^ CFU/mL, corresponding to an optical density (OD) of 0.4 at 600 nm (Chandrasekaran et al., 2017), and applied to the seedling stem base. The test was performed on 18 d-growth seedlings, applying two milliliters of *Bacillus* isolates or *B. subtilis* QST713 (Serenade^®^) suspensions at 1 × 10^8^ CFU/mL. After 40 d inoculation, plant height, stem diameter, root length, leaf number, leaf area (Canopeo app (https://canopeoapp.com)), and leaves, stem, and root dry biomass were determined. The Dickson Quality Index (DQI) was calculated using the Dickson, Leaf & Hosner (1960) formula as follows:

\begin{eqnarray*}DQI= \frac{\text{Total dry weight of the plant}\text{(g)}}{ \frac{\text{Height (cm)}}{\text{Diameter at root neck}\text{(mm)}} + \frac{\text{Shoot dry weight}(g)}{\text{Root dry weight}\text{(g)}} } \end{eqnarray*}

#### Pepper growth promotion by formulated Bacillus spp. (second experiment)

In the seedling emergence assay, we used 10-d growth Jalapeño pepper seedlings in polystyrene trays with 20 cavities, filled with sterile peat moss (1 h at 120 °C and 15 lb/in^2^ pressure) (Cosmo Peat^®^; Cosmocel, S.A., Mexico Agricultural Division). Treatments were inoculated five times at 7-d intervals, immersing trays (drench) with the microgranular formulations F-BC26, F-BC08, and F-BT24 (1 × 10^8^ CFU/mL) or *B. subtillis* (BactoRacine-B^®^ MycoBiosfera, Jalisco, Mexico) (1 × 10^7^ CFU/mL). Seedlings were watered every third day with a nutrient solution containing 10% N, 8% P_2_O_5_, 18% K_2_O, 2.5% S, 1.8% Mg, 5.9% Ca, 0.1% Fe, 0.002% B, 0.01% Zn, 0.0002% Cu, 0.0002% Mn (Nutritive Solution for Vegetables^®^, Comercializadora Hydroenviroment S.A. de C.V. México) and adjusted to pH 5.5 and 1.5 mS EC. On day 40 after inoculation, plant height, stem diameter, root length, leaf area (Canopeo app), and fresh and dry biomass of leaves, stems, and roots were determined.

F-BC26, F-BC08, and F-BT24 microgranules were applied to pepper plants in the vegetative stage. For this, 35-d-old jalapeño pepper seedlings were transplanted into 20 cm diameter polystyrene pots filled with peat moss and horticultural perlite mixture at a 3:1 ratio (vol/vol). Formulations were applied five times at intervals of 10 d from the transplant time. To achieve this, 50 mL of formulated suspensions (1 × 10^8^ CFU/mL), 50 mL of BactoRacine-B^®^ (1 × 10^7^ CFU/mL), or 50 mL of nutrient solution were applied to each plant stem base. All plants were watered every third day with nutrient solution (Nutrient Solution for Vegetables^®^; pH 5.5 and 1.5 mS EC). After 85 d of first inoculation (transplant time), plant height, stem diameter, root length, leaf area, the number of leaves, and fresh and dry biomass of leaves, stems, and roots were determined. Carotenoids, chlorophyll “a”, and chlorophyll “b” content were also determined (Lichtenthaler & Wellburn, 1983).

### *In vitro* antagonism of *Bacillus* spp. versus *Xanthomonas* spp.

*In vitro Xanthomonas* growth inhibition by *Bacillus* isolates was determined in microplates, using 22 *Bacillus* strain isolates as treatments (antagonists). In addition, *B. subtilis* QST 713 (Serenade^®^) was evaluated as the control antagonist, *X. euvesicatoria* Xp47 and Xe65 (Cepario Lab MAFFP, UACH) as pathogens, and *X. campestris* ATTC1395 as the negative control. Bacterial suspensions were prepared from 24 h growth cultures at 28 °C and 120 rpm in Luria Bertani (LB) liquid medium at pH 7.0. Bacterial density was adjusted to 1 ×10^8^ CFU/mL, corresponding to 0.4 at 600 nm OD by UV-visible spectrophotometry (Evolution 60 S; Thermo Fisher Scientific Inc., Waltham, MA, USA) for *Xanthomonas* and *Bacillus* spp. (Rabbee, Ali & Baek, 2019). Bacterial suspensions were placed in a microplate, adding 75 µL of LB-cultured *Xanthomonas* per well plus 75 µL of *Bacillus* suspensions. We used 150 µL of LB as an absolute control, whereas as a positive control, we tested 75 µL of LB plus 75 µL of each phytopathogenic bacterium. Microplates were sealed with parafilm^®^ and incubated at 28 °C for 72 h. *Xanthomonas* growth was confirmed every 24 h by reseeding, using the streak plate technique in NA and incubating at 28 °C for 48 h. *Xanthomonas* growth inhibition by *Bacillus* was qualitatively determined, considering, as a positive result, the presence of the antagonistic effect of *Bacillus*. For this, the following arbitrary scale was used: +, regular; ++, good; and +++, intense, when no pathogen colonies were observed in the reseeding in NA at 24 h, 48 h, and 72 h of confrontation. It was considered a negative result (absence of *Bacillus* antagonistic effect) when the growth of at least one colony of the pathogen was detected in the reseeding, during the evaluation period (the “-” sign indicates no growth inhibition).

### Resistance induction in pepper against *Xanthomonas*

We evaluated plant resistance to *Xanthomonas* by F-BC26, F-BC08, and F-BT24 strains. This test was established in a plant growth room with a photoperiod of 16 h light at 28 °C and 8 h dark at 18 °C and 80% to 90% RH. In this assay, we used 35-d-old jalapeño pepper plants variety M. Seedlings were cultivated in 10 cm diameter pots with sterile peat moss and irrigated every third day with the nutrient solution proposed by Otazu (2010). Plants were inoculated during transplantation time at stem base level, using 10 mL of F-BC26, F-BC08, or F-BT24 ( 1 × 10^8^ CFU/mL) suspensions, whereas 10 mL of Serenade^®^ (1 × 10^8^ CFU/mL) or nutrient solution (FitoFort^®^ (15.9% P, 21.5% K, 1.5% Zn, 1.7% sulfur, 1.6% Mn, and 58.7% plant extracts))

(Fruverint Comercializadora S.A de C.V., Mexico City, Mexico). Plants without any inducing treatment were used as controls. After seven days, plants foliage (two points on two leaves per plant) was inoculated through infiltration with one milliliter Plastipak SFP syringes (Becton-Dickinson, Brooklyn, NY), using 10 µL of *X. euvesicatoria* Xp47 and Xe65 suspensions at 1 × 10^8^ CFU/mL or 0.85% sterile saline solution (control). Before and after pathogens inoculation, plants were conditioned for two days in darkness, with an RH greater than 90%. Disease inhibition by inducing resistance was determined after 15 d of pathogens inoculation, using the Nutter, Esker & Netto (2006) modified formula as follows:

\begin{eqnarray*}\text{Disease inhibition}(\text{%})= \frac{1-\text{Disease severity in leaf infiltrated phytobacterial formulation}}{\text{Disease severity in control}} \times 100 \end{eqnarray*}

Where: $\text{disease severity} \left( \text{%} \right) = \frac{\text{disease leaf area}}{\text{total leaf area}} \times 100$

### Foliar biocontrol of *Xanthomonas*

In this assay, we used 35-d-old jalapeño pepper plants variety M. Seedlings were cultivated under the same conditions as for the resistance bioassay mentioned above. For biocontrol testing, three milliliters of treatment suspensions were applied by foliage spraying. Treatments consisted of F-BC26, F-BC08, or F-BT24 strain suspensions (1 × 10^8^ CFU/mL) plus 0.005% Tween 20, Serenade^®^ (as a positive control at 1 × 10^8^ CFU/mL), 10 mL of 50% Anglosan CL^®^ (didecyldimethylammonium chloride (DDAC); American Pharma S.A. de C.V.), 10 mL of FitoFort^®^ applied to the stem base (as resistance inducer), or 10 mL of sterile distilled water. After 48 h, approximately three milliliters of *X. euvesicatoria* Xp47 and Xe65 suspensions at 1 ×10^8^ CFU/mL or sterile saline solution at 0.85% were sprayed on foliage as negative controls. Before and after pathogens inoculation, plants were conditioned for two days in darkness, with a RH higher than 90%. *Xanthomonas* spp. biocontrol by *Bacillus* spp. was evaluated after 15 d of pathogen inoculation and *Bacillus* positive antagonistic effect was recorded if disease symptoms were not observed in leaves, whereas *Bacillus* absence of antagonistic effect was recorded when disease symptoms on the foliage were observed. In addition, pathogen presence in leaf tissue was determined by taking samples, grinding them with a pestle, and taking microdilutions to inoculate on MacConkey Agar plates (Lugo et al., 2012).

### Statistical analysis

Pepper growth promotion by *Bacillus* spp. test in the seedlings emergence was established under a completely randomized design, where 22 isolated strains from soil were tested as treatments, Serenade^®^ as a positive control, and seedlings without PGPB as control. All treatments had five replicate determinations. Pepper growth promotion by formulated *Bacillus* spp. tests in the seedlings emergence assay and the vegetative stage were established under a completely randomized design, where F-BC26, F-BC08, and F-BT24 formulations were tested as treatments, BactoRacine-B^®^ as positive control, and seedlings or plants without formulations as controls. Seedlings emergence assay and vegetative stage assays had four and five replicate determinations per treatment, respectively.

*In vitro* antagonism of *Bacillus* spp. *versus Xanthomonas* spp. the test was established under a completely randomized design. The 22 *Bacillus* isolated strains from soil were evaluated as treatments, Serenade^®^ as a positive control, and *X. euvesicatoria* Xp47, *X. euvesicatoria* Xe65, and *X. campestris* ATTC1395 as the control group. All treatments had three replicate determinations.

Resistance induction in pepper against *Xanthomonas* spp. and foliar biocontrol of *Xanthomonas* spp. by formulated *Bacillus* spp. tests were established under a completely randomized design, where F-BC26, F-BC08, and F-BT24 formulations were tested as treatments, Serenade^®^, FitoFort^®^, and Anglosan^®^ CL (only foliar biocontrol test) as a positive control group, and *X. euvesicatoria* Xp47 and *X. euvesicatoria* Xe65 as the negative controls, and plants without formulations as controls. Resistance induction assays were performed five times, whereas foliar biocontrol experiments had four replicate determinations per treatment.

Data from *Bacillus-* induced pepper plants growth with unformulated and formulated *Bacillus* spp. strains were analyzed by ANOVAs, and the Scott-Knott’s (*α* = 0.05) and the Tukey’s (*α* = 0.05) mean separation tests, respectively. Data from *Xanthomonas* spp. biocontrol assays were analyzed by ANOVAs and Tukey’s (*α* = 0.05) mean separation test. All analyzes were performed with the InfoStat software (InfoStat version 2009; InfoStat Group, Cordoba, Argentina).

## Results

### Isolation and identification of *Bacillus* spp.

**Figure 1:**
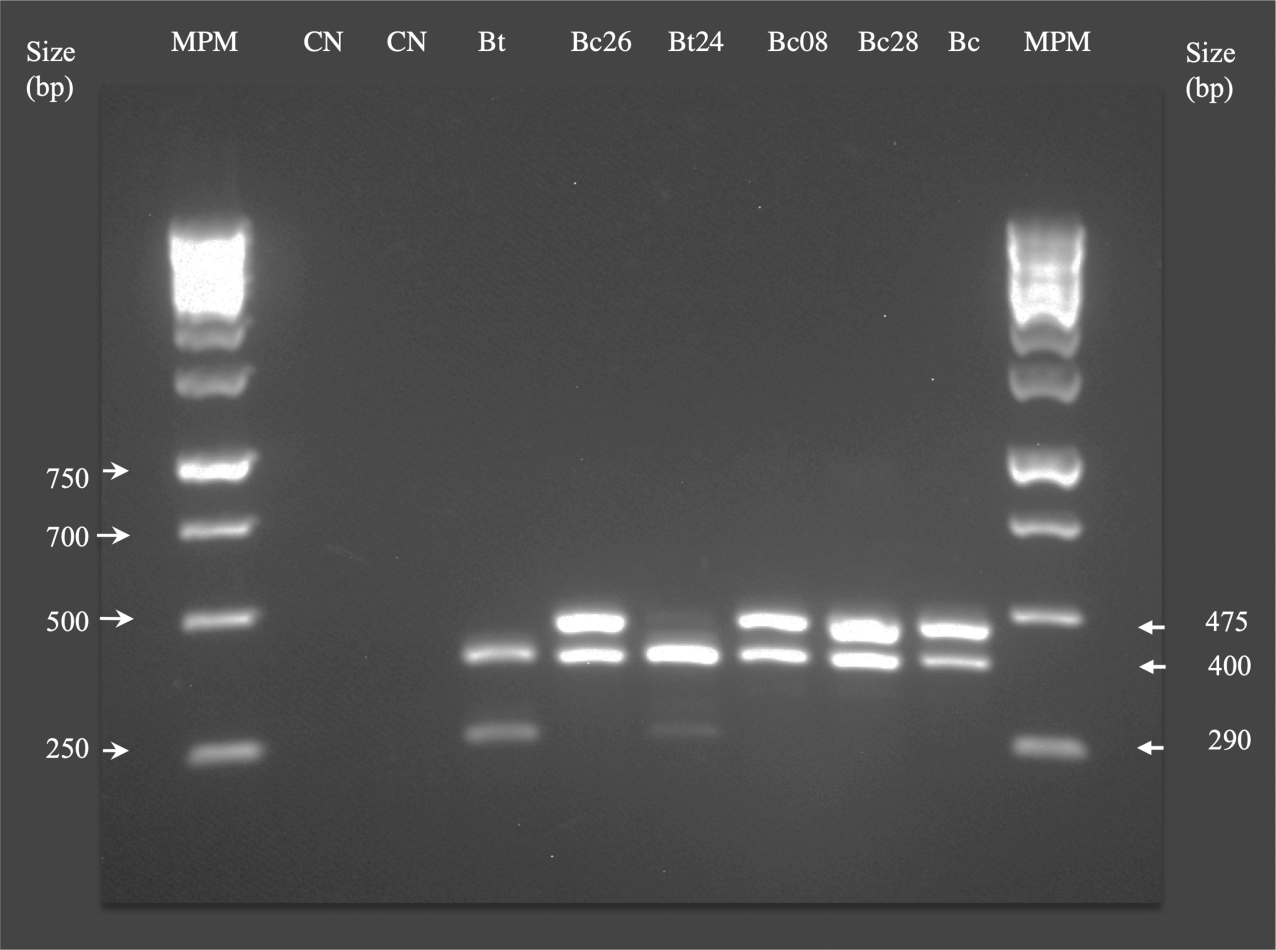
*Bacillus* spp. molecular identification by PCR. Agarose gel testing for specific primer pairs for *Bacillus* spp. Lanes: MPM = 120 bp low range DNA ladder molecular weight marker; NC, negative control; Bs, *Bacillus subtilis*; Bt, *Bacillus thuringiensis* (400 and 299 bp); Bc, *Bacillus cereus*; (475 bp and 400 bp), and Bc26 to Bc28 = isolates.

Samples exposure to 90 °C is recommended for selecting mostly *Bacillus* spp. isolates. This step is recommended since these temperature range does not affect spore survival, but helps to eliminate other undesirable bacteria. After exposing samples at 90 °C by 10 min, mostly viable *Bacillus* spp. endospores will develop in the culture medium (Cohan, Roberts & King, 1991). Processing soil samples at such conditions, allowed the isolation and purification of 22 bacterial strains with morphological characteristics expected for the genus *Bacillus*. Macroscopic analysis showed that bacteria grew in large, circular shapes, wavy edges, opaque and smooth textures, and low elevation colonies, which also presented colors with variations between light gray or creamy to whitish. Microscopically, bacteria showed bacillary form, were Gram-positive, and positive to Schaeffer-Fulton stain, thus indicating endospores presence. Strains were positive for motility and negative to the KOH test. Most *Bacillus* spp. isolates were molecularly identified by multiplex PCR as *B. cereus* (21 isolates) and only one as *B. thuringiensis*. Isolates identification was confirmed with the 400 bp and 299 bp DNA fragments amplification of one isolate, using *B. thuringiensis*-specific primers, and 400 bp and 475 bp fragments amplification in isolates testing *B. cereus*-specific primers (Fig. 1). No amplification was observed for negative controls and other primer pairs specific for *B. anthrasis*, *B. licheniformis*, *B. mycoides*, or *B. subtilis*.

### *Bacillus* spp. as pepper PGPB

Pepper seedlings growth under controlled conditions significantly (*P* < 0.05) improved after *Bacillus* spp. application (seedling emergence assay) (Table 2). After individual application of 22 isolates in seedlings, seven isolates increased leaves area by 46.8%, and 12 increased leaves number by 11.4%, as compared with the absolute control. In addition, seedling height improved between 14.2% and 28.8%, as compared with the absolute control seedlings, after applying 20 of the strains. After application, 21 out of 22 *Bacillus* spp. strains, improved pepper stem diameter between 9.9% and 27.8%, as compared with the control seedlings. Furthermore, 11 out of 22 strains, showed 8.8% higher root length, compared with the control (Table 2).

**Table 2:** *Bacillus* spp. **effect** on jalapeño pepper seedlings growth^1^.

Treatments	LA (cm^2^ plant^−1^)	LN (plant^−1^)	PH (cm)	SD (mm)	RL (cm)
Control-fertilizer	34.2 ± 6.2^b^	8.9 ± 0.9^b^	13.5 ± 1.5^c^	1.8 ± 0.2^c^	7.8 ± 1.1^b^
Serenade^®^	41.0 ± 7.2^b^	8.9 ± 1.4^b^	17.3 ± 2.1^a^	2.5 ± 0.4^a^	8.3 ± 1.7^a^
*B. cereus* CBC01	48.1 ± 7.0^a^	9.6 ± 0.8^a^	15.3 ± 1.3^b^	1.1 ± 0.2^d^	7.8 ± 1.0^b^
*B. cereus* CBC02	41.5 ± 5.3^b^	9.3 ± 1.7^b^	16.5 ± 1.4^a^	1.1 ± 0.3^d^	7.4 ± 1.0^b^
*B. cereus* CBC05-3	42.5 ± 11.3^b^	9.9 ± 1.3^a^	17.2 ± 2.2^a^	2.1 ± 0.3^b^	6.8 ± 0.9^b^
*B. cereus* CBC05-4	37.8 ± 8.3^b^	8.9 ± 1.1^b^	14.7 ± 1.2^b^	2.2 ± 0.3^b^	7.8 ± 0.7^b^
*B. cereus* CBC05-5	43.4 ± 4.6^b^	9.6 ± 0.5^a^	15.6 ± 1.7^b^	2.1 ± 0.3^b^	8.7 ± 1.3^a^
*B. cereus* CBC07-3	40.0 ± 13.1^b^	8.5 ± 1.1^b^	12.7 ± 2.8^c^	2.2 ± 0.4^b^	8.2 ± 1.3^a^
*B. cereus* CBC07-5	50.8 ± 15.2^a^	9.2 ± 0.6^b^	14.8 ± 1.5^b^	1.4 ± 0.3^b^	8.1 ± 1.1^a^
*B. cereus* CBC08	49.4 ± 9.2^a^	10.1 ± 0.7^a^	17.6 ± 1.4^a^	2.2 ± 0.2^b^	9.0 ± 1.7^a^
*B. cereus* CBC09-4	39.7 ± 13.1^b^	9.8 ± 1.4^a^	15.3 ± 1.6^b^	2.1 ± 0.2^b^	8.2 ± 1.5^a^
*B. cereus* CBC13	46.9 ± 8.1^b^	10.0 ± 0.8^a^	19.2 ± 2.2^a^	2.2 ± 0.2^b^	7.1 ± 1.3^b^
*B. cereus* CBC15-3	43.6 ± 9.3^b^	9.0 ± 0.9^b^	15.6 ± 2.2^b^	2.1 ± 0.3^b^	7.1 ± 0.7^b^
*B. cereus* CBC19	42.8 ± 8.7^b^	10.5 ± 1.4^a^	15.6 ± 2.1^b^	2.3 ± 0.3^a^	8.1 ± 1.4^a^
*B. cereus* CBC21-2	43.5 ± 7.8^b^	9.2 ± 0.9^b^	15.9 ± 1.4^b^	2.1 ± 0.2^b^	7.7 ± 1.1^b^
*B. cereus* CBC21-5	43.9 ± 5.8^b^	9.7 ± 1.4^a^	12.6 ± 4.0^c^	2.1 ± 0.3^b^	8.7 ± 1.4^a^
*B. cereus* CBC24-3	42.9 ± 1.3^b^	8.9 ± 1.2^b^	14.7 ± 1.2^b^	1.1 ± 0.3^d^	8.3 ± 1.5^a^
*B. thuringuiensis* CBT24	42.0 ± 8.0^b^	9.5 ± 1.1^a^	15.8 ± 1.7^b^	2.1 ± 0.3^b^	7.8 ± 1.2^b^
*B. cereus* CBC25	49.1 ± 9.8^a^	9.4 ± 1.3^b^	17.1 ± 2.0^a^	2.0 ± 0.4^b^	9.1 ± 1.2^a^
*B. cereus* CBC26-1	40.8 ± 6.2^b^	9.4 ± 0.8^b^	16.6 ± 0.9^a^	2.1 ± 0.3^b^	7.6 ± 0.7^b^
*B. cereus* CBC26	55.0 ± 9.8^a^	10.2 ± 1.1^a^	17.4 ± 1.9^a^	2.3 ± 0.2^a^	7.4 ± 1.2^b^
*B. cereus* CBC28	44.5 ± 8.2^b^	9.4 ± 1.5^b^	15.3 ± 1.6^b^	2.2 ± 0.2^b^	8.4 ± 2.1^a^
*B. cereus* CBC29-3	49.4 ± 6.9^a^	9.7 ± 1.3^a^	17.2 ± 1.8^a^	2.1 ± 0.3^b^	8.6 ± 1.1^a^
*B. cereus* CBC29-4	49.7 ± 8.1^a^	10.0 ± 0.9^a^	15.9 ± 3.1^b^	2.1 ± 0.1^b^	7.8 ± 1.6^b^

**Notes.**

1 LA, leaf area; LN, Leaves number; PH, plant height; SD, stem diameter; and RL, root length. Data represent means ± SD of two plants by replicate (five replicate determinations). Each value in columns followed by different letters indicates significant (*P* <0.05) difference by ANOVA and the Scott Knott test.

The evaluated strains (3, 7, and 13 of 22) increased the dry weight of roots, stems, and leaves by 46.1%, 45.4%, and 37.8%, respectively, as compared with the absolute control seedlings and commercial product. Regarding seedlings quality, expressed in terms of the Dickson Index, CBC08 and CBC26 strains improved plant quality by 60.7%, as compared with the absolute control seedlings (Table 3). Overall, 15 of all tested strains induced the same quality of seedlings as the commercial product treatment (Table 3).

**Table 3:** *Bacillus* spp. **effect** on jalapeño pepper seedlings development^1^.

**Treatments**	**RW (g)**	**SW (g)**	**LW (g)**	**DQI**
Control-fertilizer	0.048 ± 0.01^b^	0.174 ± 0.05^b^	0.114 ± 0.03^b^	0.025 ± 0.01^b^
Serenade^®^	0.050 ± 0.02^b^	0.193 ± 0.06^b^	0.114 ± 0.04^b^	0.027 ± 0.01^b^
*B. cereus* CBC01	0.052 ± 0.02^b^	0.258 ± 0.06^a^	0.153 ± 0.04^a^	0.021 ± 0.01^c^
*B. cereus* CBC02	0.050 ± 0.02^b^	0.224 ± 0.06^b^	0.136 ± 0.03^b^	0.018 ± 0.01^c^
*B. cereus* CBC05-3	0.045 ± 0.02^b^	0.156 ± 0 .07^b^	0.100 ± 0.03^b^	0.021 ± 0.01^c^
*B. cereus* CBC05-4	0.042 ± 0.02^b^	0.191 ± 0.07^b^	0.107 ± 0.05^b^	0.024 ± 0.01^b^
*B. cereus* CBC05-5	0.056 ± 0.02^b^	0.220 ± 0.09^b^	0.156 ± 0.05^a^	0.030 ± 0.01^b^
*B. cereus* CBC07-3	0.031 ± 0.02^b^	0.148 ± 0.06^b^	0.104 ± 0.05^b^	0.020 ± 0.01^c^
*B. cereus* CBC07-5	0.051 ± 0.02^b^	0.256 ± 0.09^a^	0.169 ± 0.06^a^	0.025 ± 0.01^b^
*B. cereus* CBC08	0.084 ± 0.03^a^	0.283 ± 0.07^a^	0.180 ± 0.05^a^	0.040 ± 0.01^a^
*B. cereus* CBC09-4	0.053 ± 0.02^b^	0.220 ± 0.08^b^	0.144 ± 0.08^a^	0.028 ± 0.01^b^
*B. cereus* CBC13	0.054 ± 0.02^b^	0.260 ± 0.06^a^	0.152 ± 0.04^a^	0.028 ± 0.01^b^
*B. cereus* CBC15-3	0.065 ± 0.02^a^	0.246 ± 0.06^a^	0.161 ± 0.04^a^	0.034 ± 0.01^b^
*B. cereus* CBC19	0.047 ± 0.01^b^	0.170 ± 0.05^b^	0.110 ± 0.04^b^	0.026 ± 0.01^b^
*B. cereus* CBC21-2	0.055 ± 0.02^b^	0.219 ± 0.04^b^	0.131 ± 0.02^b^	0.029 ± 0.01^b^
*B. cereus* CBC21-5	0.041 ± 0.01^b^	0.222 ± 0.07^b^	0.143 ± 0.04^a^	0.027 ± 0.01^b^
*B. cereus* CBC24-3	0.050 ± 0.02^b^	0.216 ± 0.09^b^	0.140 ± 0.06^a^	0.021 ± 0.01^c^
*B. thuringuiensis* CBT24	0.050 ± 0.02^b^	0.184 ± 0.06^b^	0.116 ± 0.04^b^	0.026 ± 0.01^b^
*B. cereus* CBC25	0.059 ± 0.02^b^	0.204 ± 0.06^b^	0.141 ± 0.03^a^	0.027 ± 0.01^b^
*B. cereus* CBC26-1	0.059 ± 0.02^b^	0.218 ± 0.06^b^	0.132 ± 0.04^b^	0.029 ± 0.01^b^
*B. cereus* CBC26	0.073 ± 0.02^a^	0.308 ± 0.05^a^	0.195 ± 0.04^a^	0.040 ± 0.01^a^
*B. cereus* CBC28	0.052 ± 0.02^b^	0.209 ± 0.07^b^	0.124 ± 0.04^b^	0.029 ± 0.01^b^
*B. cereus* CBC29-3	0.056 ± 0.02^b^	0.231 ± 0.07^b^	0.145 ± 0.03^a^	0.029 ± 0.01^b^
*B. cereus* CBC29-4	0.054 ± 0.01^b^	0.257 ± 0.05^a^	0.161 ± 0.03^a^	0.031 ± 0.01^b^

**Notes.**

1 RW, root dry weight; SW, stem dry weight; LW, leaf dry weight; and DQI = Dickson Quality Index. Data represent means ± SD of two plants by replicate (five replicate determinations). Each value in columns followed by different letters indicates significant (*P* <0.05) difference by ANOVA and the Scott Knott test.

Pepper seedlings grown under greenhouse conditions (seedlings emergence assay), significantly (*P* < 0.05) improved after applying formulated *B. cereus* and *B. thuringiensis* (Table 4). Formulated F-BT24 strain improved the stem diameter and root length by 16.2% and 10.2%, respectively, as compared with the un-inoculated control seedlings and commercial product. Both formulations separately applied, improved seedling height, leaf area, and stem and leaves dry weights by 46.1%, 36.9%, 38.1% and 39.1%, respectively, as compared with the absolute control seedlings. Formulated F-BC08 strain only improved height by 25.5% and induced comparable results as the commercial treatment and the control in the remaining assayed parameters.

**Table 4:** Formulated *Bacillus* spp. effect on jalapeño pepper seedlings growth and development under greenhouse conditions.

Treatments	SD (mm)	PH (cm)	LA (cm^2^ plant^−1^)	RL (cm)	LW (g)	SW (g)	RW (g)
Control	1.25 ± 0.19^b^	6.61 ± 1.98^c^	16.5 ± 6.3^b^	9.16 ± 2.38^ab^	0.0232 ± 0.011^b^	0.0098 ± 0.0038^b^	0.0095 ± 0.0014^a^
BactoRacine	1.24 ± 0.19^b^	7.12 ± 1.74^c^	17.9 ± 4.3^b^	8.09 ± 2.23^b^	0.0258 ± 0.007^b^	0.0109 ± 0.0032^ab^	0.0070 ± 0.0022^a^
F-BC26	1.33 ± 0.14^b^	9.80 ± 0.73^a^	22.3 ± 3.6^a^	8.84 ± 2.31^ab^	0.0326 ± 0.006^a^	0.0134 ± 0.0025^a^	0.0096 ± 0.0020^a^
F-BC08	1.26 ± 0.10^b^	8.62 ± 0.83^b^	18.0 ± 4.8^b^	8.10 ± 2.01^b^	0.0393 ± 0.006^a^	0.0123 ± 0.0011^ab^	0.0079 ± 0.0018^a^
F-BT24	1.45 ± 0.18^a^	9.52 ± 0.66^a^	22.8 ± 3.7^a^	9.50 ± 2.04^a^	0.0356 ± 0.010^b^	0.0137 ± 0.0038^a^	0.0101 ± 0.0026^a^
*LSD*	0.10037	0.80075	2.84204	1.34382	0.03081	0.00349	0.00421

**Notes.**

1 SD, stem diameter; PH, plant height; LA, leaf area; RL, root length; LW, leaf dry weight; SW, stem dry weight; and RW, root dry weight. *LSD*, Least Significant Difference. Data represent means ± SD of ten plants by replicate (four replicate determinations). Each value in columns followed by different letters indicates significant (*P* <0.05) difference by ANOVA and the Tukey test.

**Figure 2:**
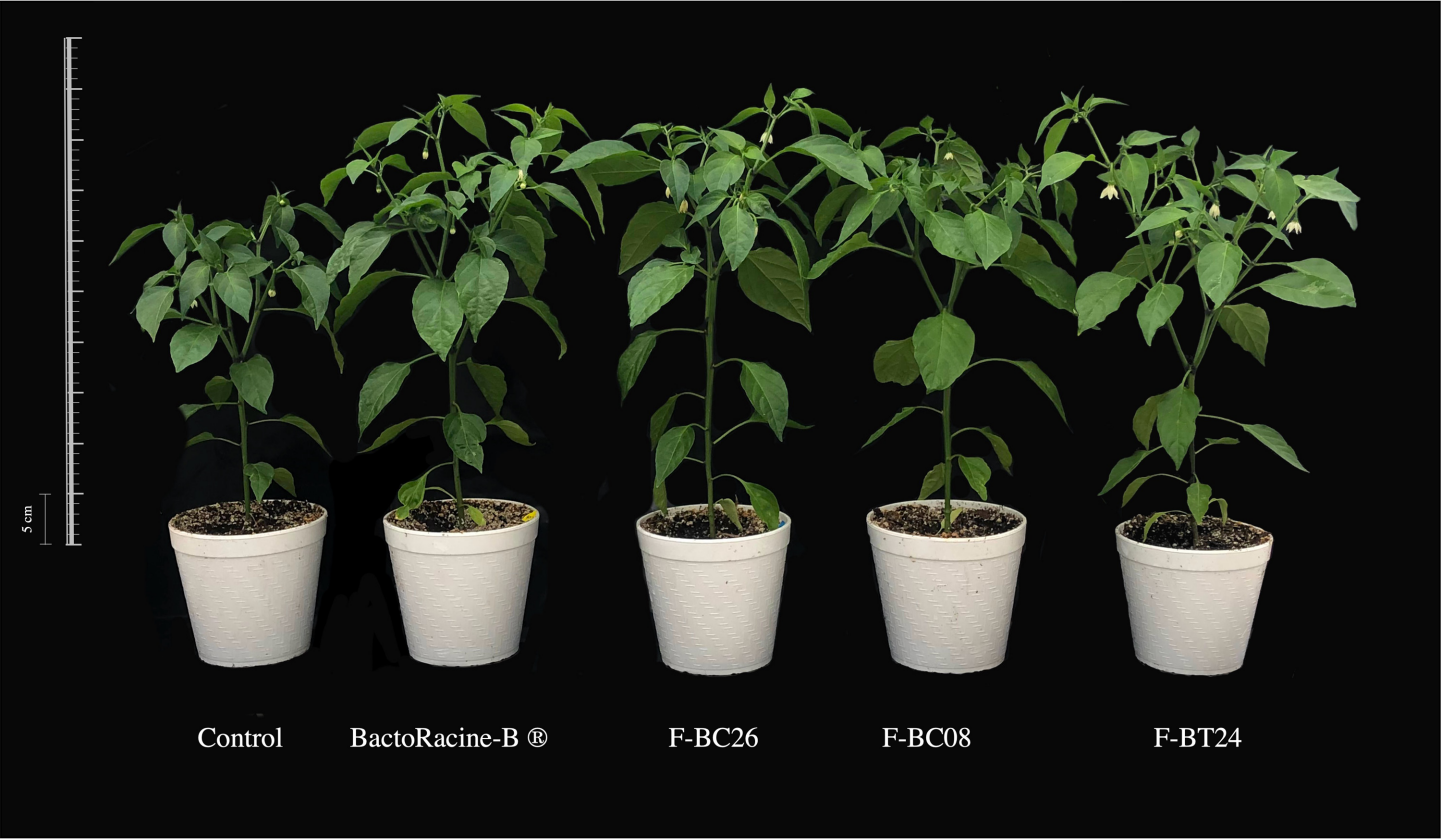
Formulated *Bacillus* spp. effect on jalapeño pepper plants under greenhouse conditions. Control = fertilized plants, BactoRacine^®^ = growth promoter commercial product based on *Bacillus subtilis*, F-BC08 and F-BC26 = formulated *B. cereus*; and F-BT24, formulated *B. thuringiensis*. All treatments were fertilized.

Potted pepper plants development under greenhouse conditions (vegetative stage) was significantly higher (*P* <  0.05) after applying formulated *B. cereus* and *B. thuringiensis* strains, as compared with fertilized plants (Table 5 and Fig. 2). The three tested formulations and the commercial product significantly (*P* < 0.05) improved leaf area, stem diameter, and leaf and stem dry weights by 82.0%, 20.6%, 62.9%, and 72.7%, respectively, as compared with the absolute control. F-BC26, F-BT24, and commercial product treatments, increased height and root dry weight by 48% and 141%, respectively, as compared with the absolute control. Furthermore, F-BC26 formulation and the commercial product improved the number of leaves by 41.1%, whereas only the F-BT24 formulation enhanced root length by 30.6%. Similarly, F-BC08 formulation improved chlorophyll “b” and carotenoid content by 157.7% and 33.0%, respectively, as compared with the commercial product. Regarding flowering stimulation, formulated F-BC26 strain significantly increased the number of flowers to 140.7%, as compared with fertilized plants (Table 5 and Fig. 2).

**Table 5:** Formulated *Bacillus* spp. effect on jalapeño pepper seedlings growth and development of potted jalapeño pepper under greenhouse conditions^1^.

**Treatments**	**Control**	**BactoRacine**	**F-BT24**	**F-BC26**	**F-BC08**	*LSD*
NBF	14.8 ± 1.5^b^	29.5 ± 9.1^ab^	33.3 ± 8.3^ab^	35.5 ± 14.6^a^	29.8 ± 6.2^ab^	19.677
LN (plant^−1^)	44.8 ± 2.5^b^	64.8 ± 5.3^a^	62.8 ± 5.2^ab^	65.0 ± 14.0^a^	61.5 ± 9.7^ab^	18.314
SD (mm)	4.5 ± 0.2^b^	5.4 ± 0.3^a^	5.4 ± 0.3^a^	5.6 ± 0.4^a^	5.3 ± 0.2^a^	0.6295
PH (cm)	32.8 ± 1.7^b^	49.3 ± 3.2^a^	50.0 ± 5.1^a^	45.8 ± 4.3^a^	42.8 ± 7.5^ab^	10.780
LW (g)	1.95 ± 0.1^b^	3.33 ± 0.2^a^	3.05 ± 1.2^a^	3.27 ± 0.6^a^	3.06 ± 0.2^a^	1.0629
SW (g)	2.2 ± 0.2^b^	4.0 ± 0.3^a^	3.9 ± 0.4^a^	3.8 ± 0.4^a^	3.5 ± 0.6^a^	0.9188
RW (g)	0.5 ± 0.1^b^	1.1 ± 0.1^a^	1.0 ± 0.4^a^	1.1 ± 0.2^a^	0.9 ± 0.2^ab^	0.4634
LA (cm^2^ plant^−1^)	417.2 ± 39.8^b^	796.1 ± 124.7^a^	746.4 ± 123^a^	771.1 ± 124^a^	722.9 ± 47^a^	218.47
RL (cm)	18.4 ± 0.5^b^	21.4 ± 2.5^ab^	24.0 ± 2.0^a^	22.8 ± 3.9^ab^	22.0 ± 1.4^ab^	5.1246
Chla (mg/g.gfw^−1^)	1.02 ± 0.12^a^	0.92 ± 0.19^a^	1.06 ± 0.26^a^	1.16 ± 0.11^a^	1.27 ± 0.04^a^	0.3582
Chlb (mg/g.gfw^−1^)	0.54 ± 0.18^ab^	0.32 ± 0.08^b^	0.57 ± 0.34^ab^	0.53 ± 0.19^ab^	0.82 ± 0.25^a^	0.4911
Caroteniods (mg/g.gfw^−1^)	0.62 ± 0.03^ab^	0.48 ± 0.10^b^	0.59 ± 0.09^ab^	0.59 ± 0.03^ab^	0.64 ± 0.01^a^	0.1387

**Notes.**

1 NBF, buds’ flowers numbers; LN, Numbers of leaves; SD, stem diameter; PH, plant height; LW, leaf dry weight; SW, stem dry weight; RW, root dry weight; LA, leaf area; RL, root length; Chla, chlorophyl “a”; Chlb, chlorophyl “b”; F-BC08 and F-BC26, formulated *Bacillus cereus*; and F-BT24, formulated *B. thuringiensis*. *LSD*, Least Significant Difference. Data represent means ± SD of four replicate determinations. Each value in columns followed by different letters indicates significant (*P* <0.05) difference by ANOVA and the Tukey test.

### *In vitro Bacillus* spp. antagonism versus *Xanthomonas* spp

All *Bacillus* isolates tested inhibited *in vitro X. euvesicatoria* Xp47 and Xe65 growth after 24 h of confrontation (Table 6). However, only *X. campestris* ATTC1395 was inhibited by *Bacillus* spp. after 24 h of confrontation (63% inhibition), but at 48 h, its growth was completely inhibited (Table 6).

**Table 6:** *In vitro* Xanthomonas growth inhibition by *Bacillus* isolates.

**Antagonist** ^1^	**Pathogens**								
	*X. campestris* ATTC1395			***X. euvesicatoria* Xp47**			***X. euvesicatoria*** Xe65		
	24 h	48 h	72 h	24 h	48 h	72 h	24 h	48 h	72 h
*Control*	–	–	–	–	–	–	–	–	–
*B. subtilis* QST 713	–	++	+++	-	++	+++	–	++	+++
*B. cereus* CBC01	–	++	+++	+	++	+++	+	++	+++
*B. cereus* CBC02	+	++	+++	+	++	+++	+	++	+++
*B. cereus* CBC05-3	+	++	+++	+	++	+++	+	++	+++
*B. cereus* CBC05-4	+	++	+++	+	++	+++	+	++	+++
*B. cereus* CBC05-5	+	++	+++	+	++	+++	+	++	+++
*B. cereus* CBC07-3	–	+	++	+	++	+++	+	++	+++
*B. cereus* CBC07-5	–	+	++	+	++	+++	+	++	+++
*B. cereus* CBC08	–	+	++	+	++	+++	+	++	+++
*B. cereus* CBC09-4	+	++	+++	+	++	+++	+	++	+++
*B. cereus* CBC13	+	++	+++	+	++	+++	+	++	+++
*B. cereus* CBC15-3	+	++	+++	+	++	+++	+	++	+++
*B. cereus* CBC19	+	++	+++	+	++	+++	+	++	+++
*B. cereus* CBC21-2	+	++	+++	+	++	+++	+	++	+++
*B. cereus* CBC21-5	–	+	++	+	++	+++	+	++	+++
*B. cereus* CBC24-3	+	++	+++	+	++	+++	+	++	+++
*B. thuringuiensis* CBT24	+	++	+++	+	++	+++	+	++	+++
*B. cereus* CBC25	+	++	+++	+	++	+++	+	++	+++
*B. cereus* CBC26-1	+	++	+++	+	++	+++	+	++	+++
*B. cereus* CBC26	+	++	+++	+	++	+++	+	++	+++
*B. cereus* CBC28	–	+	++	+	++	+++	+	++	+++
*B. cereus* CBC29-3	–	+	++	+	++	+++	+	++	+++
*B. cereus* CBC29-4	–	+	++	+	++	+++	+	++	+++

**Notes.**

1 (+ = regular; ++ = good; +++ = intense) = *In vitro Xanthomonas* growth inhibition (nutrient agar medium); (-) = absence of *in vitro Xanthomonas* growth inhibition. Control = 0.85% saline solution.

### Resistance induction in pepper against *Xanthomonas*

**Figure 3:**
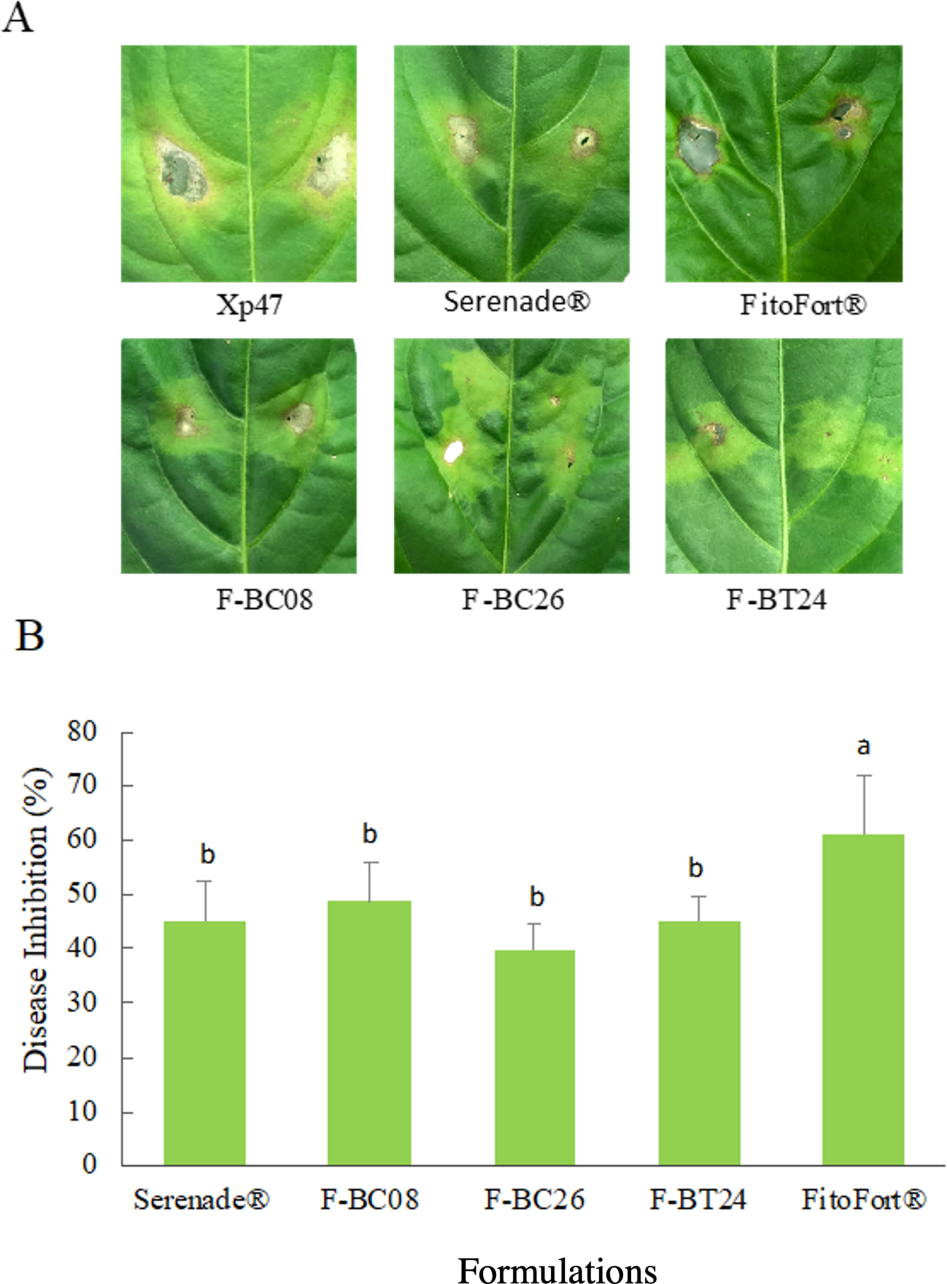
Formulated *Bacillus* spp. effect on pepper bacterial spot severity under controlled conditions, after 15 d of X. euvesicatoria (strain Xp47) inoculation as pathogen. (A) Representative signs of disease in pepper plants due to Xp47 and formulated *Bacillus* spp. (F-BC08, F-BC26, or F-BT24), Serenade^®^ (B. subtilis QST 713), or FitoFort^®^ (resistance inducer). (B) Inhibition of bacterial spot caused by *Xanthomonas euvesicatoria* Xp47 in pepper plants by *Bacillus* spp. formulations under controlled conditions. Bars with the same letter were not statistically different Tukey (*n* = 5).

Formulated *B. cereus* and *B. thuringiensis* were applied as bacterial spot resistance promoters in pepper plants under controlled conditions, showing a significant disease inhibition (*P* < 0.05) (Fig. 3). Similarly, the bacterial spot was reduced by 44.7%, after applying the commercial product Serenade^®^, whereas FitoFort^®^ induced the highest disease inhibition (61.2% disease inhibition) (Fig. 3A).

Xp47 strain in interaction with *Bacillus* formulations or commercial products showed characteristic disease signs in treated plants but lower severity depending on the treatment. Necrotic spots with chlorotic edges presence characterized them, leaves with chlorosis or perforation areas with leaf blades, and necrotic spots with chlorosis (Figs. 3A and 3B).

### Foliar *Xanthomonas* biocontrol

Bacterial spot development on pepper plants was inhibited after applying *Bacillus* spp. formulations and Serenade^®^ directly to foliage. Although it was intended for the root system, FitoFort^®^ inhibited disease development. Xp47 treated plants showed small necrotic spots on leaf blade. However, pathogen presence in foliage was found among all biological treatments, but as expected, it was not shown in the chemical treatment (Anglosan^®^ CL50%). Overall, maximum pathogen population reduction by biocontrol agents was 8.6% (Table 7).

**Table 7:** *Xanthomonas euvesicatoria* **bacterial spot inhibition in pepper plants by** ***Bacillus*** **spp.** **isolates under controlled conditions.**

**Treatments**	**Foliar signs of disease** ^1^	**Pathogen presence in foliage(Log**_**10**_UFC/cm^**2**^)
*X. euvesicatoria* Xp47	+	5.93 ± 0.03^a^
Serenade^®^	–	5.53 ± 0.10^bc^
F-BC26	–	5.85 ± 0.08^ab^
F-BC8	–	5.76 ± 0.10^ab^
F-BT24	–	5.42 ± 0.23^c^
FitoFort^®^	–	5.26 ± 0.26^c^
Anglosan^®^ CL	–	0.0 ± 0.0^d^
*L SD*		0.33750

**Notes.**

1 (+), Leaf spots presence; (-), leaf spots absence. Serenade^®^, *Bacillus subtilis* strain QST 713 commercial product for *Xanthomonas* sp. control; FitoFort^®^, commercial product to induce plant diseases resistance; F-BC26 and F-BC08, formulated *B. cereus*, F-BT24, formulated *B. thuringiensis*, and Anglosan^®^ CL, DDAC at 50%. *LSD*, Least Significant Difference. Data represent means ± SD of four replicates. Each value in columns followed by different letters indicates significant (*P* <0.05) difference by ANOVA and the Tukey test.

## Discussion

Bacterial strains isolated from soil samples in Meoqui, Chihuahua, Mexico, showed morphological characteristics of the *Bacillus* genus, as previously reported by Calvo & Zúñiga (2010), who isolated strains of the same genus but from potato rhizosphere soil. Furthermore, molecular identification confirmed that 21 strains belonged to *B. cereus* and one to *B. thuringiensis*. The presence of this type of bacterium in soils is related to its spore-forming potential, which provides resistance in different terrestrial environmental conditions, including agricultural soils (Stephens, 1998; Petersohn et al., 2001).

In the present study, we showed that *B. cereus* and *B. thuringiensis* strains application promoted pepper plants growth under greenhouse conditions, as reported by other *Bacillus* spp. such as *B. amyloliquefasciens*, *B. pumilus*, *B. subtilis,* and *B. velezensis* (Joo et al., 2004; Park et al., 2010; Son et al., 2014; Datta et al., 2015; Aguilar et al., 2017; Hernández et al., 2018; Jiang et al., 2018; Guenoun et al., 2019; Mekonnen & Fenta, 2020). This bacteria group has been reported as plant growth promoters and biocontrol agents for plant diseases by microorganisms and insect pests (Saxena, Karthikeyan & Rajawat, 2017; Kashyap et al., 2019; Tiwari, Prasad & Lata, 2019; Mahapatra, Yadav & Ramakrishna, 2022). Several studies have indicated that *Bacillus* spp. act as PGPB and biocontrol agent based on its potential to successfully colonize plant roots, by producing exo-polysaccharides, siderophores, or phytohormones, solubilizing phosphorus or fixing dinitrogen (Yanti & Nasution, 2017; Tunsagool et al., 2019; Gupta et al., 2021). Other reports have shown plants defense response against microbial phytopathogens or insect pests, which is determined by the host plant interaction (Vejan et al., 2016; Tunsagool et al., 2019).

Selected *B. cereus* and *B. thuringiensis* isolates were formulated using the spray-drying technique (F-BC26, F-BC08, or F-BT24). After liquid samples are prepared, they are “sprayed” inside of the tank where temperature is higher than 80 °C. Sample residence is of a few seconds, since it dries, and resulting micro-particles are vacuumed to a collector container. It has been proven that *Bacillus* spp. spores survive this spray drying process (Tamez-Guerra et al., 2000).

*B. cereus* and *B. thuringiensis* have been studied for their fungicidal and insecticidal activities, respectively. However, few studies report their potential as biofertilizers and biocontrol agents against phytopathogenic bacteria. Hence the importance of this study, because it evidences these bacteria potential as PGPB by improving the chili plants growth, under greenhouse conditions and control bacterial spot. Both bacterial strains increased seedling size, stem diameter, leaf area, root length, and stem and root dry weights, an effect similar to or greater than that produced by *B. velezensis, B. amyloliquefasciens,* and *B. subtilis* strains, after applying on chili plants (Mirik, Aysan & Çinar, 2008; Park et al., 2010; Datta et al., 2015; Samaniego et al., 2016; Hernández et al., 2018; Guenoun et al., 2019; Kashyap et al., 2021; Shin et al., 2021). Furthermore, chlorophyll “a” and carotenoid content in chili plants was improved by bacterial treatments. Chlorophyll “a” increase was similar to that reported by Seon et al. (2014), testing *Bacillus* sp. in chili plants. Park et al. (2010) demonstrated that chlorophyll “a” content increase induces plant growth in pepper plants due to plant metabolism changes. Similarly, carotenoids content increase has been reported in other crops after *Bacillus* sp. inoculation. Alamri et al. (2019) found that these pigments increased in lettuce plants grown under greenhouse conditions, after *B. subtilis* inoculation. In our study, pepper plants flowering increase may have been related to gibberellic acid production by *B. cereus* (Mekonnen & Kibret, 2021).

These results demonstrated that *B. cereus* and *B. thuringiensis* promoted pepper plants growth under greenhouse conditions, due to their rhizosphere colonization potential and phytohormones production, such as indole-3-acetic acid (IAA) and gibberellic acid. Previous studies have shown that *B. cereus* and *B. thuringiensis* improve pepper plants development by IAA production (Hyder et al., 2020; Jo et al., 2020). In addition, gibberellins production by *B. cereus* has been reported as a pepper growth promotion mechanism (Joo et al., 2004). Despite these bacteria benefits, there are a few products based on *B. cereus* as PGPB, and the use of *B. thuringiensis* for this purpose is limited (Azizoglu, 2019).

In our study, *in vitro Xanthomonas* growth inhibition by *Bacillus* spp. evidenced *Bacillus* spp. potential to synthesize antimicrobial secondary metabolites against this phytopathogen. The cyclic lipopeptides (CLPs) iturin-like and fengicine of the surfactin family, have been widely documented as antimicrobial compounds produced by *Bacillus* spp. (Ongena & Jacques, 2008; Raaijmakers et al., 2010; Cochrane & Vederas, 2016). CLPs antibacterial activity against phytopathogens such as *X. campestris* has been attributed to iturins and surfactins (Zhao et al., 2018) and recently to fengicines (Medeot et al., 2020). For instance, it has been reported that *B. subtilis* SSE4 produces iturins that have shown antibacterial activity against *X. campestris* (Thasana et al., 2010). Grady et al. (2019) reported that *B. velezensis* inhibited *in vitro X. campestris* and *X. euvesicatoria* growth by surfactin action. Surfactins are inserted into bacterial cell membranes, solubilizing the phospholipid bilayer and creating pores and ionic channels, causing cell death (Hamley, 2015; Zhao et al., 2018). Medeot et al. (2020) indicated that exposure to fengicines produced by *B. amyloliquefaciens* causes alterations in *X. axonopodis* pv. *vesicatoria* cell topography, which results in cell death by intracellular content filtration.

In the present study, *in vitro Xanthomonas* growth inhibition by *Bacillus* spp. may have resulted from the nutrients and habitat competition between both bacteria, since it has been reported as another biological control mechanism used by *Bacillus* spp. (Chen et al., 2020; Pedraza, López & Uribe-Vélez, 2020). Competition between species causes a reduction in growth, productivity, and other activities (Shafi, Tian & Ji, 2017). It was also observed that *Bacillus* displaced *Xanthomonas* spp. in the culture medium, which was evident upon their growth activity every 24 h, since only *Bacillus* colonies grew throughout the evaluation period, except after 24 h interactions with *X. campestris* ATCC1395.

Bacterial spot biocontrol under controlled conditions in pepper plants, through formulated *B. cereus* and *B. thuringiensis*, evidenced the ISR mechanism by these antagonists. These results are similar to those reported by Mirik, Aysan & Çinar (2008), who showed a disease reduction between 11% to 62%, after evaluating *X. axonopodis* pv. *vesicatoria* biocontrol in pepper plants under greenhouse conditions, applying *Bacillus* spp. at transplantation.

Similarly, Pajčin et al. (2020) after applying *B. velezensis* in pepper plants for *X. euvesicatoria* biocontrol found significant suppression of disease signs to up to 76%, whereas Chandrasekaran et al. (2017) after applying *B. subtilis* CBR05 on tomato plants, found a 54.4% severity reduction of bacterial spot caused by *X. vesicatory* pv. *campestris*. Yanti, Habazar & Resti (2017) detected a disease reduction between 15.76% to 42.51% by applying *B. thuringiensis* in soybeans for *X. axonopodis* pv. *glycines* biocontrol.

Our biocontrol results after direct foliage spraying with formulated *Bacillus* spp. were observed to be higher than that reported by others, who evaluated *Bacillus* for *Xanthomonas* spp. control on horticultural crops. Abdurrahman, Ahamed & Amein (2020) reported 18.2% bacterial spot severity reduction caused by *X. campestris* pv. *vesicatoria* on tomato by applying *B. subtilis* K3 suspension on tomato seedlings foliage under greenhouse conditions, whereas Hassan & Zyton (2017) observed a 6.4% severity reduction caused by *X. campestris* pv. *vesicatoria* after spraying *B. subtilis* on pepper plants in the field. Furthermore, Elsisi (2017) reported a 29.6% severity reduction of *X. campestris* pv. *campestris* on cabbage plants established in the field through *Bacillus* sp. foliar application. However, it must be considered that such results were obtained under controlled conditions, which may favor *Bacillus* spp. biocontrol mechanisms against *Xanthomonas* spp.

In this regard, the main phyllosphere biocontrol mechanism used by *Bacillus* spp. has been attributed to surfactins, iturins, and fengicines production, which synergistically function as antimicrobials and allow successful antagonist colonization (Legein et al., 2020). It has been reported that *B. subtilis* surfactins trigger biofilm formation, which allows colonization and antimicrobial compounds to release the control (Wei, Hu & Xu, 2016). Iturins and fengicines alter fungi and bacteria cell membranes, allowing their control (Raaijmakers et al., 2010; Cochrane & Vederas, 2016). Zeriouh et al. (2011) achieved a significant disease inhibition due to iturin production after foliar *B. subtilis* applications on melon leaves *in vitro* to control the melon bacterial spot caused by *X. campestris* pv*. cucurbitae*.

Another biocontrol mechanism used by *Bacillus* sp. may be the ”quorum extinction”, which consists of substances production that degrades phytopathogens signaling molecules, thus regulating their virulence (Ma et al., 2013; Legein et al., 2020). Such substances would indicate *X. euvesicatoria* Xp47 presence, which was re-isolated from the phyllosphere. Similarly, induction of plant defense mechanisms might be involved but has yet to be elucidated (Hassan & Zyton, 2017; Tyagi et al., 2018). For example, Elsisi (2017) indicated that *Bacillus* spp. application on pepper plants from cabbage established in the field, significantly increased quitinase, peroxidase, and oxidase polyphenol activities, which participate in plants defense mechanisms against phytopathogens.

## Conclusions

The present study demonstrated the potential of *B. cereus* (21 strains) and *B. thuringiensis* (CBT24) as PGPB, when applied during the emergence of pepper seedlings and crop development under greenhouse conditions. Formulations of F-BT24 and F-BC26 strains significantly promoted pepper seedlings growth and crop development. *In vitro* testing demonstrated that Bacillus isolates showed antagonistic potential against *X. euvesicatoria*. Under controlled condition experiment, F-BT24, F-BC26, and F-BC08 formulations efficiently controlled bacterial spot, similar to the commercial product (Serenade^®^). Based on these results, formulated *B. cereus* and *B. thuringiensis* may be used as biofertilizers and biocontrol agents against chili peppers bacterial spot. However, it is necessary to continue studying and evaluating their effects on yield and fruit quality.

##  Supplemental Information

10.7717/peerj.14633/supp-1 Supplemental Information 1 Raw data used for the statistical analysis of each parameter and to create Table 1 to Table 6 and Fig. 2

## Additional Information and Declarations

### Field Study Permissions

The following information was supplied relating to field study approvals (i.e., approving body and any reference numbers): Field experiments were approved by the Fundación Produce, Chihuahua, A.C.

### Data Availability

The following information was supplied regarding data availability: The raw measurements are available in the Supplementary File.
